# Explosion Suppression Mechanism Characteristics of MEMS S&A Device With In Situ Synthetic Primer

**DOI:** 10.3390/mi9120652

**Published:** 2018-12-10

**Authors:** Hengzhen Feng, Wenzhong Lou, Dakui Wang, Fuquan Zheng

**Affiliations:** National Key Laboratory of Electro-Mechanics Engineering and Control, School of Mechatronical Engineering, Beijing Institute of Technology, Beijing 100081, China; louwz@bit.edu.cn (W.L.); wangdakui2018@gmail.com (D.W.); han0718@bit.edu.cn (F.Z.)

**Keywords:** MEMS safety and arming device, explosion suppression mechanism, energy absorbing material, explosion suppression mechanism reliability

## Abstract

The traditional silicon-based micro-electro-mechanical systems (MEMS) safety and arming (S&A) device fuze cannot isolate abnormal outputs in the detonation environment, which creates hazards for personnel. To address this problem, we report the design of a MEMS S&A device with integrated silver, copper, nickel and polyimide (PI) films, which is based on the principle of a MEMS S&A device and uses copper azide as the primer. The MEMS S&A device was optimized using theoretical calculations of the explosion suppression mechanism performance in a detonation field, where the theoretical model was verified by dynamic simulation (LS-Dyna). Silicon-based MEMS processing technology was used to integrate the MEMS S&A device with energy-absorbing materials, and the device performance was compared in detonation tests. Silicon-based MEMS S&A devices with silver, copper, nickel, and PI (100-μm-thick) achieved a reliable explosion suppression mechanism capability when exposed to a detonation wave. The residual stress was measured using Raman microscopy, and the PI film exhibited the best explosion suppression mechanism performance of the four materials. A reliability test to determine the maximum explosion suppression mechanism dose for a MEMS S&A device attached to a PI film (100-μm-thick) showed that the maximum amount of primer needed for the effective explosion suppression mechanism capability on the MEMS S&A device was 0.45 mg.

## 1. Introduction

The micro-electro-mechanical systems (MEMS) safety and arming (S&A) device has the advantages of a small size, a light-weight mass and a high reliability, which reduces the structural size significantly and provides space for additional functionality [1,2,3]. Therefore, the MEMS S&A device has become a highly researched topic [4,5]. The US Navy has made significant progress in the research of MEMS S&A devices since Robinson [6] proposed the “Lithographie, Galvanformung and Abformung” (i.e., lithography, electroplating and molding, or lithographie galvanoformung (LIGA) and abformung) process in 1998 and fabricated the first MEMS S&A device that complied with environmental-protection requirements. The main design concept of the nickel or copper MEMS S&A device developed by Robinson et al. was its modular characteristics, where LIGA or ultraviolet LIGA processes could be used to achieve integrated processing of some functional modules, such as the integration of a MEMS-based safe arm and fire device [7,8]. However, springs fabricated by LIGA separately can raise the cost and make it difficult to assemble. Steven S. Mink [9] and Robert A.Lake used an electro-thermal principle to actuate the SA device, and successfully minimized its size into millimeters utilizing surface micromachining technology. Another design idea for the MEMS S&A device is the high-density integration of mechanical, electronic and pyrotechnic technologies. Based on silicon materials, deep-reactive-ion etching (DRIE) or silicon-on-insulator processes were used to achieve integrated processing of MEMS S&A devices. For example [10], the MEMS S&A device designed at the Indian Head Division of the Naval Surface Warfare Center was processed on silicon via DRIE to yield a safety mechanism and firing components that could be used in various environments. In this device, the core functions of safety and the explosion-suppression-mechanism control could be achieved at a large scale and at low cost. The use of an in-plane V-shaped electric heat driver as a remote release mechanism allowed for integration and mass production. In 2014, Wang Fufu et al. invented a MEMS security-system structure [11] that is now used primarily for fuze safety systems. In their design, the safety system is placed perpendicular to the fuze spring axis and double safety is used [12,13], whereby the system is designed to have the protective function of release from a long distance. Although this MEMS S&A device exhibits a high reliability and stability and a low power consumption [14,15], because of its special base materials it possesses a poor explosion-suppression-mechanism performance and cannot absorb the energy generated by the explosion-suppression mechanism [16,17]. In addition, the detonator is a device which can generate a high speed slapper to trigger the explosion [15]. Therefore, we studied the explosion-suppression-mechanism performance of a silicon-based MEMS S&A device and characterized its reliability by using theoretical calculations, static dynamic simulations and experimental verification.

## 2. MEMS Safety and Arming (S&A) Device Design

**Figure 1:**
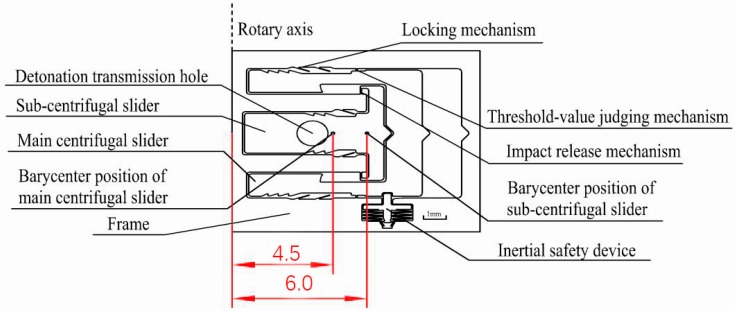
The MEMS safety and arming device (S&A) overall structure.

A schematic of the designed silicon-based MEMS S&A device used (11 mm × 10 mm × 0.4 mm) is shown in Figure 1. The MEMS S&A device is used mainly for rotary ammunition. The bary center position of the main centrifugal slider and the sub-centrifugal slider, and the position of the projectile rotary axis are shown in Figure 1.

As shown in Figure 1, the silicon-based MEMS S&A device was placed parallel to the rotary axis. Under normal conditions, when ammunition is launched, the spring moves downwards under traction, and the inertial safety device is released. Because of the centrifugal force, the main centrifugal slider and sub-centrifugal slider start to move along the centrifugal force direction, and the explosion train is aligned. However, in an abnormal situation, the explosion train is not aligned, the primer is triggered, and the main centrifugal slider and sub-centrifugal slider need to detonate energy effectively. In addition, the centrifugal threshold-determination mechanism is weak, so the impact resistance of the threshold-determination mechanism is key to the integrated MEMS S&A device.

We simulated the anti-overload capability of the centrifugal threshold-judgment mechanism of the MEMS S&A device parametrically, and the model was simplified with a relative position of the threshold-judgment mechanism, the main centrifugal slider and the sub-centrifugal slider. The frame of the MEMS S&A device was restrained and the centrifugal acceleration mode was set by ANSYS pre-processing. After LS-dyna post-processing, the maximum stress output of the MEMS S&A device under a centrifugal condition was obtained, which yielded the main centrifugal slider, sub-centrifugal slider and the centrifugal threshold-determining-mechanism parametric design.

**Figure 2:**
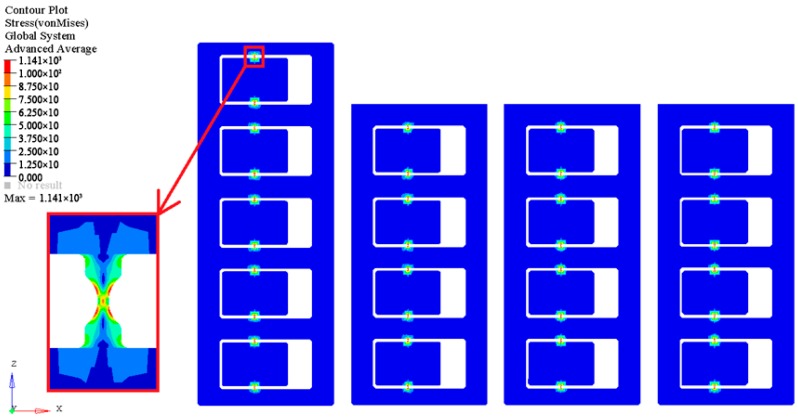
Stress of the centrifugal overload of the MEMS S&A device.

The MEMS S&A device model used simulates the system dynamically as a function of the centrifugal environment (Figure 2). In a centrifugal environment of 315,000 m^2^/s, the main centrifugal slider and sub-centrifugal slider move and the threshold-determining mechanism is broken. The device strength is 1.14 GPa, which meets the design requirements of a launch environment.

Figure 2 provides simulation results for the stress in the parameterized threshold value connection units. During the repeated experiments, the g-values for fracture of the threshold value judging mechanisms from the same fabrication batch and with the same design parameters present a certain degree of discreteness, which shows that a certain degree of discreteness exists in the mechanical properties of the silicon material. Therefore, this discreteness of the mechanical properties of the silicon material should be considered in the design process.

## 3. Parametric Calculation Method of MEMS S&A Device Under Denotation Field

When the MEMS S&A device is safe and the stimulant is ignited accidentally, if the MEMS S&A device cannot be exploded reliably, it will impact the MEMS fuze accidentally and may cause great harm. Therefore, the explosion-suppression-mechanism performance of the MEMS S&A device must be studied. A fluid–solid coupling algorithm is used mainly to study the dynamic response of MEMS S&A devices in the explosion-suppression-mechanism field [12].

### 3.1. Calculation Method of Explosion-Suppression-Mechanism Performance of MEMS S&A Device

**Figure 3:**
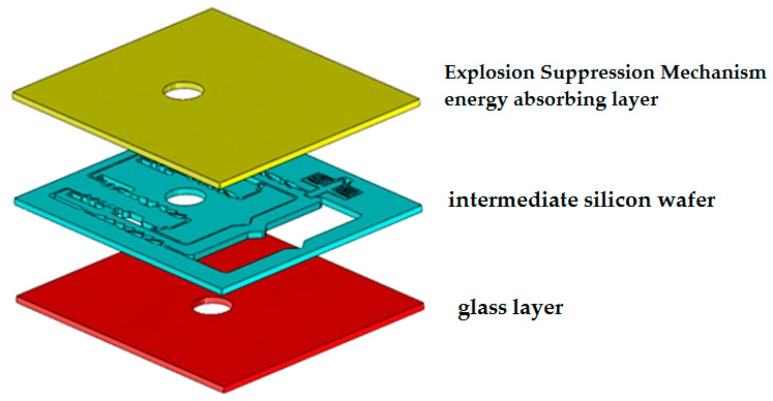
The silicon-based MEMS S&A device.

The silicon-based MEMS S&A device consists of an explosion-suppression-mechanism energy-absorbing layer, a glass layer and an intermediate silicon wafer, with the structure shown in Figure 3. When the blasting holes of the glass layer and the silicon layer are misaligned, the denotation wave on the denotation energy-absorbing layer can ignite unexpectedly, and the denotation wave will impact the silicon wafer. The glass, silicon wafer and denotation energy-absorbing layer thicknesses were 300 μm, 400 μm and 100 μm, respectively, and the material parameters of the Si, metals and polyimide (PI) used are given in Table 1.

The detonation mode is integrated into the metal bridge wire and copper azide. The inductive-detonation wave output of the in-situ synthesis of copper azide is achieved by the electrothermal effect of a metal bridge wire. In the detonation field environment, the basic formula for the detonation wave pressure is given as:

(1) $$p=Fpeos\left(V_{1},E\right)$$

where *peos* is the pressure equation of the state, which is calculated from the Jones–Wilkins–Lee (JWL) state equation; *V*_1_ is the specific volume and *E* is the specific energy of the material. The parameter *F* is calculated as follows:

(2) $$\left\{ \begin{array}{l} F=\max\left(F1,F2\right)\\ F1=\{\begin{array}{ll} \left[2\left(t-t1\right)DA_{\varepsilon \max}\right]/3V_{\varepsilon } & t>t1\\ 0 & t\leq t1 \end{array}\\ F2=\left(1-V_{1}\right)/1-V_{CJ} \end{array}\right.$$

where *t* is time, *t*1 is the explosion-suppression-mechanism end time closest to the explosion-suppression-mechanism point, *D* is the explosion-suppression-mechanism velocity of the explosive, *A_εmax_* is the maximum cross-sectional area of the explosion-suppression-mechanism unit, *V_ε_* is the explosion-suppression-mechanism unit volume, and *V_CJ_* is the specific value of the Chapman–Jouguet (CJ) point.

The high-energy explosive material model must be used with the JWL state equation. The basic formula for the material pressure in the JWL state equation is given as:

(3) $$p=A\left(1-\frac{\omega^{*}}{R_{1}V}\right)e^{-R_{1}V}+B\left(1-\frac{\omega^{*}}{R_{2}V}\right)e^{-R_{2}V}+\frac{\omega^{*}E}{V}$$

where *V* is the relative volume or the ratio of the volume of the explosion-suppression-mechanism product to the volume of the unexploded explosive and *A*, *B*, *R*_1_, *R*_2_, and ω* are material constants. The material parameters of the copper azide primer and the material pressure state-equation parameters used are given in Table 2.

**Figure 4:**
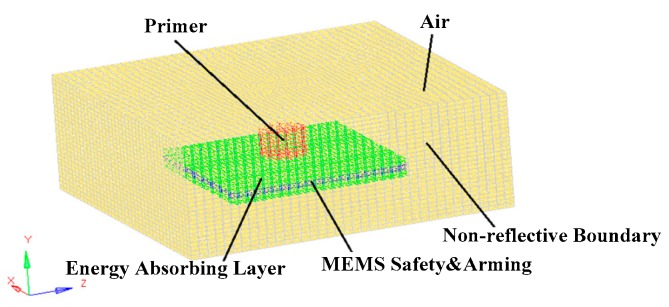
Finite element model of the MEMS S&A device under the denotation field.

**Figure 5:**
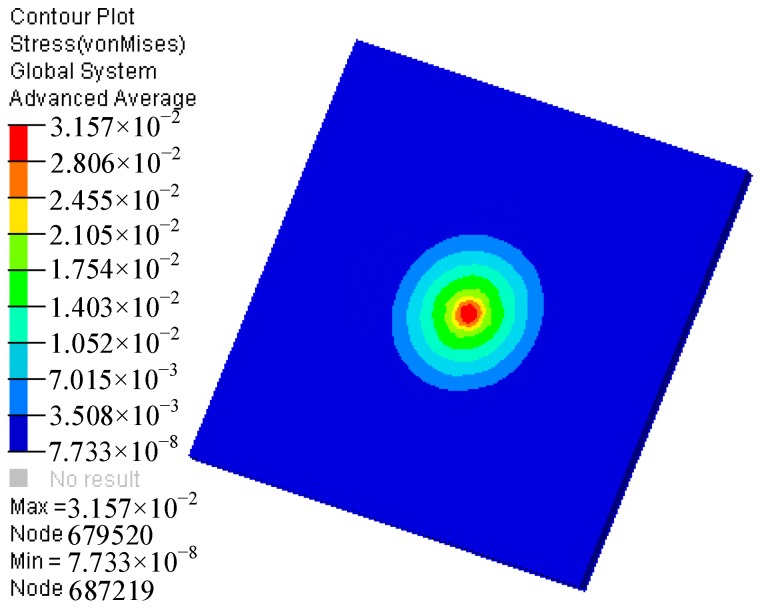
Maximum stress cloud of the MEMS S&A device.

During assembly, the primer of the MEMS S&A device was placed directly above the booster hole and near the MEMS S&A device. The primers filled the electric detonator with a mass of 0.4 mg, a diameter of 2.5 mm and a 1.47-mm length. A solid element of an arbitrary Lagrangian–Eulerian single-point integration was used to establish a computer-aided engineering model of the air and the primer. To improve the calculation speed, the explosion-suppression-mechanism module of the MEMS S&A device was plated with an equivalent. To improve the calculation accuracy, a non-reflective boundary was applied to the outer surface of the air model to simulate an infinite air lattice. The finite-element model of the established explosion-suppression-mechanism field is shown in Figure 4. The dynamic response of the MEMS S&A device in the denotation field was simulated according to the simulation conditions, and the maximum stress of the MEMS S&A device was obtained, as shown in Figure 5.

Figure 5 shows that the maximum stress on the silicon wafer is 3157 MPa, which is greater than the Allowable strength of the material itself (Table 1). Silicon is a brittle material that will break with no plastic deformation when the stress exceeds the allowable strength of the material itself. The silicon wafer did not break in a denotation field, and the 0.4 mg equivalent copper azide primer was not detonated effectively. When the primer is ignited accidentally, however, there is a serious risk of injury or death to personnel. Therefore, the improvement of the explosion suppression mechanism performance of silicon-based MEMS S&A devices has become a critical issue.

### 3.2. Parametric Optimization Design of MEMS S&A Device

To improve the explosion suppression mechanism capability of the MEMS S&A device, we parametrically modeled the copper azide primer to explore the maximum amount of copper azide primer needed to effectively detonate the silicon-based MEMS S&A device. The quality parameter for each material dimension of the parameterized copper azide primer used herein is given in Table 3.

**Figure 6:**
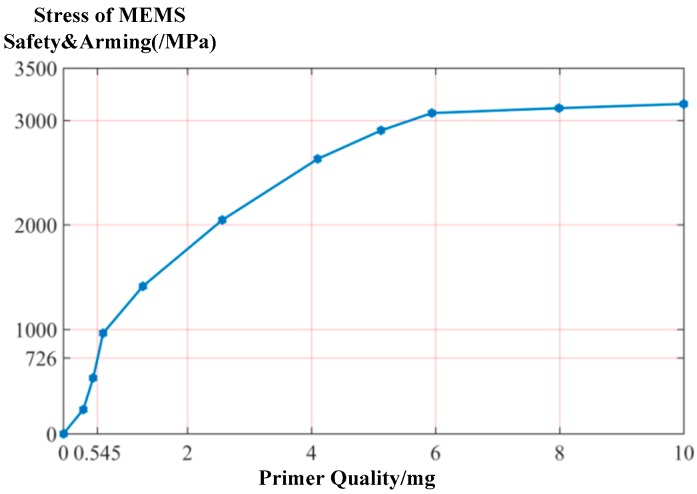
Relation between the maximum stress of the silicon wafers in the denotation field and the amount of copper azide primer.

A parameterization simulation analysis of the explosion suppression mechanism capability of the silicon-based MEMS S&A device was carried out in descending order of the parameterized copper-azide catalyst. The simulation results were used to determine the relationship between the maximum stress of the silicon wafer in the explosion suppression mechanism field and the amount of copper-azide primer, as shown in Figure 6.

A comparison of the relationship between the maximum stress of the silicon wafer in the denotation field and the amount of copper-azide primer shows that the maximum stress on the silicon wafer is correlated positively with the amount of copper-azide primer. Specifically, when the primer dose is less than 5.94 mg, the maximum stress on the silicon wafer increases sharply with an increase in the amount of primer; and when the primer dose exceeds 5.94 mg, the maximum stress of the wafer tends towards a stable value. By using the material properties of the silicon wafer, a silicon wafer-breaking strength of 726 MPa can be obtained. Therefore, the maximum amount of explosive material that can detonate the MEMS S&A device ranges between 0.38 mg and 0.55 mg, and is estimated to be 0.45 mg.

Based on these results, the MEMS S&A device was optimized to improve its explosion suppression mechanism performance. Because of the limitations of the MEMS fuze space, the structural dimensions of the MEMS S&A device cannot be changed significantly. However, metal materials possess the characteristics of plastic deformation and the capacity for absorption of the explosion suppression mechanism wave energy, so that the MEMS S&A device surface can be plated with a metal film to ensure that the overall dimensions of the structure are substantially unchanged. Layer-deposition and chemical-vapor-deposition (CVD) methods can improve the overall system reliability. We used a 100-μm PI film and copper, nickel and silver metal plating as research objects.

**Figure 7:**
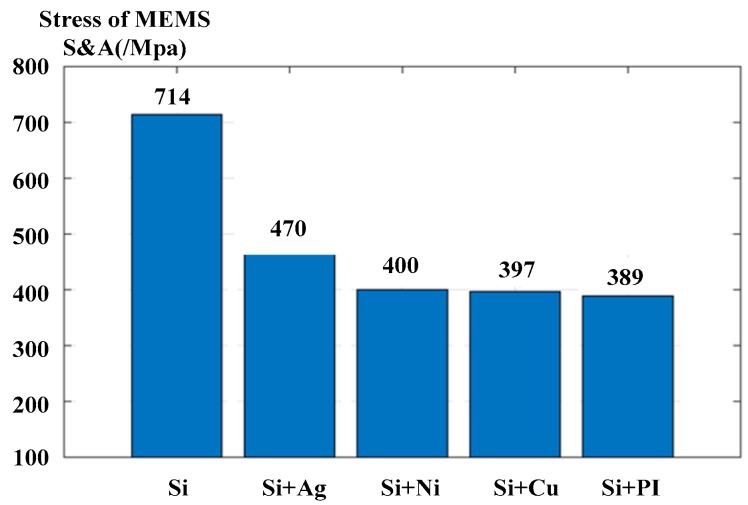
Maximum stress of the MEMS S&A device for various explosion suppression mechanism energy-absorbing materials.

For a constant primer amount (0.4 mg), the dynamic-response characteristics of the MEMS S&A device with varying explosion suppression mechanism energy-absorbing materials on the surface in an explosion suppression mechanism field were studied. The maximum stress of the MEMS S&A device with various energy-absorbing materials was obtained via simulation, as shown in Figure 7.

According to Figure 7, compared with the polished silicon wafer, surface plating of the silicon wafer with metal and a PI film can absorb the energy of the denotation wave more effectively. The maximum stress on the silicon wafer with electroplated copper, silver, nickel and with a PI film is reduced sequentially as silver > nickel > copper > PI. The PI film exhibits the lowest maximum stress because the modulus of elasticity of the PI is larger than that of the three metals, and thus the energy absorbed by the same deformation under the denotation wave is greater for the PI film.

**Figure 8:**
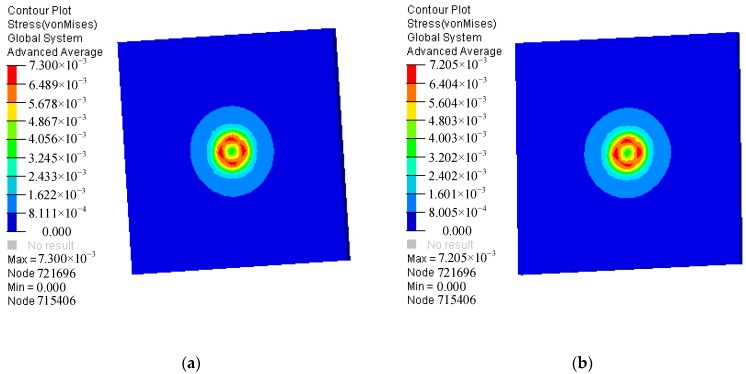
Maximum stress cloud image of a silicon wafer prepared by MEMS S&A device using a copper azide primer amount of (**a**) 0.429 mg and (**b**) 0.415 mg.

We studied the amount of copper azide primer required for the explosion suppression mechanism for the MEMS S&A device that comprised a PI film, where the simulation results are shown in Figure 8. For 0.429 mg and 0.415 mg primer, the maximum stresses on the silicon wafer are 730 MPa and 720 MPa, respectively. By combining this result with the breaking strength of the silicon material (726 MPa), it can be inferred that the maximum explosive charge of the MEMS S&A device that comprised a PI film was 0.4 mg, which is compared with the effective explosion suppression mechanism dose of 0.45 mg of the die. Therefore, the explosion suppression mechanism capability of the safety system increased by 124% with the use of a PI film. These results show that PI film deposition on the MEMS S&A device surface is an effective method to improve its anti-explosion performance.

## 4. Integrated Processing

### 4.1. Silicon-Based MEMS Safety-System Processing Technology

**Figure 9:**
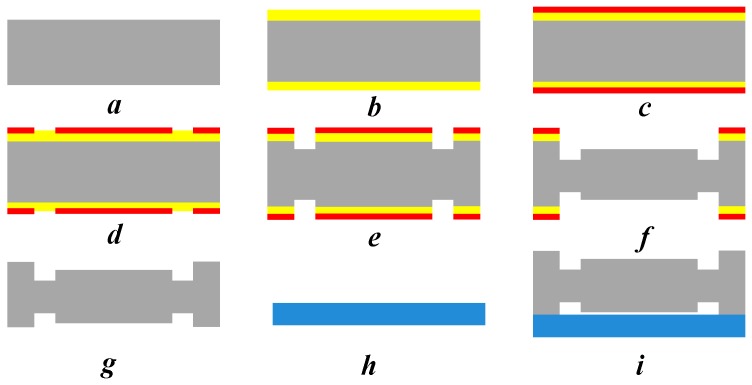
The processing flow of the threshold determination mechanism. (**a**) Preparation of 4-inch silicon wafer; (**b**) oxidation, thermal oxygen to form SiO_2_; (**c**) homogenizing; (**d**) double-sided lithography, threshold-determination-mechanism graphic selection; (**e**) deep-reactive-ion etching (DRIE)–graphical threshold-determining mechanism by DRIE; (**f**) removal of mask layer of flame-proof slider (photoresist, silicon dioxide), double-sided etching, completion of threshold-determination mechanism and explosion-proof slider processing; (**g**) removal of mask layer. Concentrated deposition of 98% sulfuric acid and hydrogen peroxide in a water bath at 120 °C to remove photoresist and physical deposition of impurities, hydrogen fluoride (HF) to remove SiO_2_ mask layer; (**h**) glass preparation; (**i**) anode bonding–glass is bonded to the silicon-based MEMS S&A device.

The silicon-based MEMS S&A device threshold-judgment mechanism and the explosion-proof slider were processed with the process flow in Figure 9.

**Figure 10:**
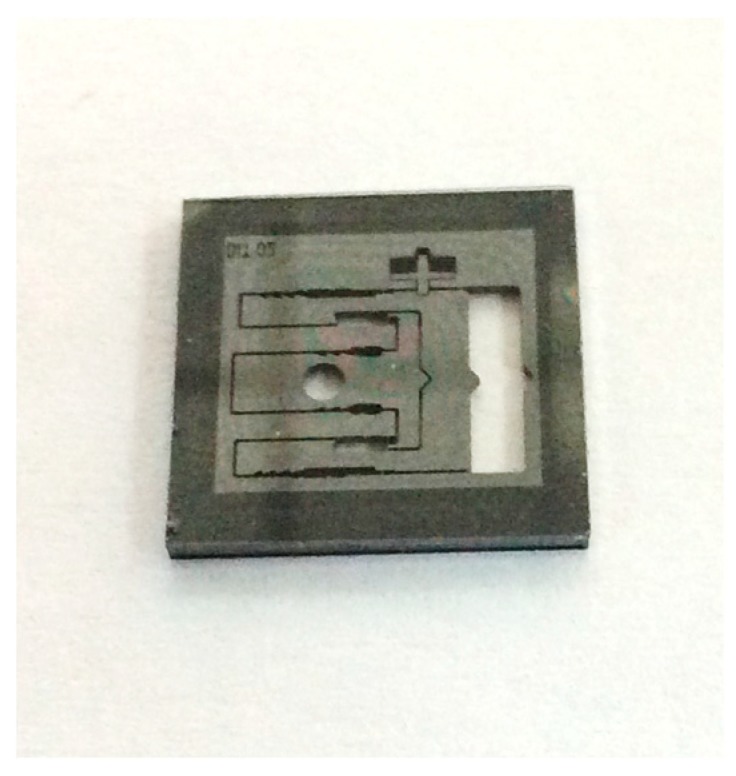
The silicon-based MEMS S&A device.

The silicon-based MEMS S&A device was shown in Figure 10. 

The silicon-based MEMS S&A device was integrated with various explosion suppression mechanism energy-absorbing materials of Ag, Cu, Ni, and PI films through magnetron sputtering, CVD and the electroplating process.

### 4.2. Electroplated Metal as Explosion Suppression Mechanism Energy Absorbing Material

**Figure 11:**
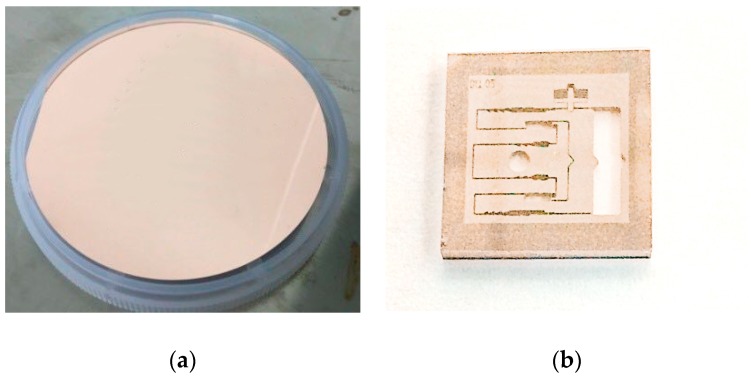
The MEMS S&A device after the Cu electroplating process. (**a**) Electroplated Cu wafer; (**b**) electroplated Cu graphics.

The explosion suppression mechanism energy-absorbing silver, copper, and nickel metals were sputtered on the MEMS S&A device surface and electroplating was used to prepare the absorbing material. We include only copper electroplating, although the processes for silver and nickel were similar. We used CuSO_4_ for the electroplating solution, and the electroplated substrate sheet was a 4-inch silicon wafer that was prepared previously by sand blasting. The electroplating process was as follows:

(1) Owing to the presence of impurities such as dust and particles, bubbles are highly likely to form during electroplating. To prevent bubble formation, the silicon substrate sheet was cleaned organically, mainly by acetone cleaning at 40 °C for 10 min and alcohol cleaning at 40 °C for 10 min. The copper seed layer was magnetron sputtered onto a substrate sheet on the silicon substrate. A bubble-prevention cleaning step was not required if the sputtering and plating times were short.
(2) The MEMS S&A device was adhered to the cathode carrier plate with copper-conductive tape and the Si was washed with high-pressure deionized water for 15 min to eliminate any bubbles inside the deep hole.
(3) The Si was soaked in 8% to 10% dilute sulfuric acid for 10 min to eliminate copper oxides on the copper seed-layer surface.
(4) The cathode stage that carried the Si was immersed vertically in CuSO_4_ plating solution for electroplating, with the electroplating parameters given in Table 4.
(5) The device was cleaned using deionized water to rinse the plated glass surface for 15 min.
(6) To reduce the high metal surface roughness of the electroplated surface, the electroplated surface was polished by a polishing machine. This polishing step is vital for the subsequent metal-patterning process. Figure 11 shows the MEMS S&A device after the Cu electroplating process.

Lithography was used to select the threshold value-determining mechanism and the flameproof slider graphic position. The explosion suppression mechanism energy-absorbing layer was patterned by wet etching as shown in Table 5. 

### 4.3. Preparation of PI Film as Denotation Energy Absorbing Material

The PI film was prepared by spin coating, with the process flow given in Table 6.

**Figure 12:**
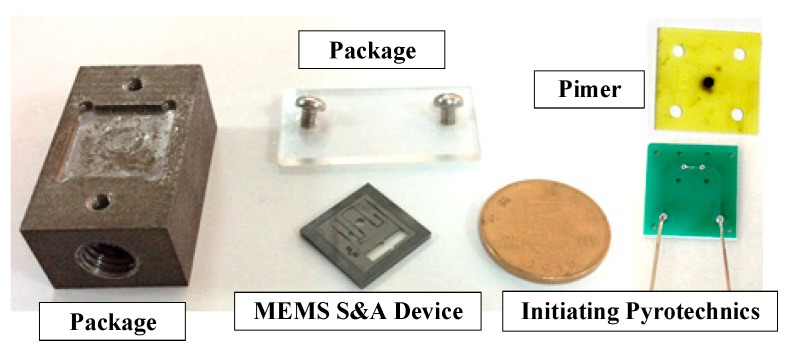
Integrated MEMS S&A device.

Using the processes specified in Section 4.1 and Table 6, the metal film and PI preparation were completed and the MEMS S&A device was integrated with the denotation energy-absorption layer. The integrated MEMS S&A device is shown in Figure 12.

## 5. Explosion Suppression Mechanism Test

**Figure 13:**
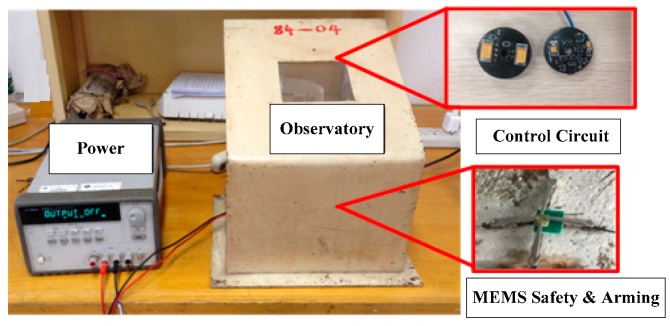
Test platform used to test the den energy-absorbing materials.

**Figure 14:**
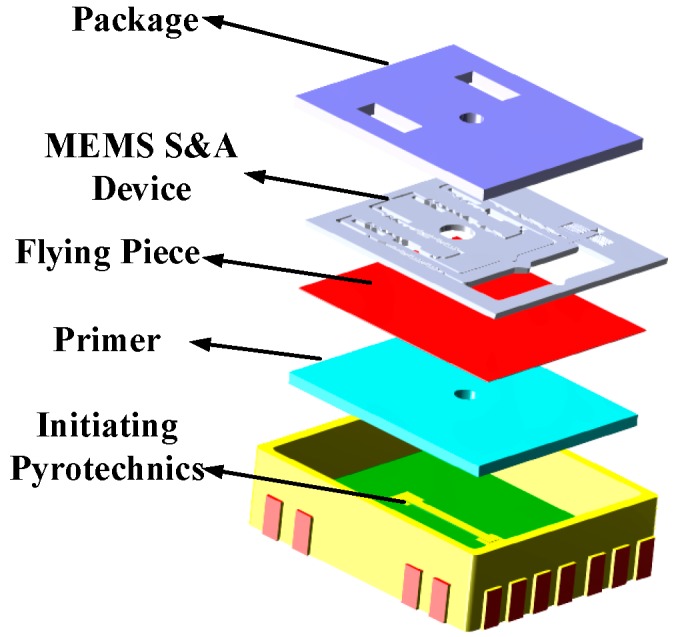
Integrated explosion train.

We constructed an explosion suppression mechanism system test to verify the abilities of the various denotation energy-absorbing materials to absorb the denotation energy and to achieve a reliable explosion suppression mechanism performance of the MEMS S&A device. The test platform consisted of a power supply, a delay control module, a MEMS S&A device with an energy-absorbing material, a MEMS S&A device without an energy-absorbing material, and a microcharge system (Figure 13). An exploded view diagram that shows all of the elements of the test samples is given in Figure 14.

**Figure 15:**
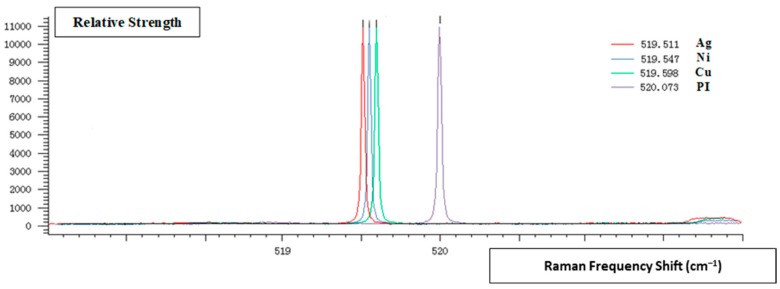
Raman Spectroscopy Residual Stress.

The delay control module governs the system detonation delay by setting details for the detonation. Its test process is as follows:

(1) The power supply was engaged to power the delay control module system.
(2) The delay control module was set to delay the detonation command by 30 s.
(3) When the delay time was complete, the delay control module output the detonation signal, which detonated the integrated microcharge system. The residual stress of the MEMS S&A device was tested by observing if the system in the explosion suppression mechanism state possessed breaks or chips. The explosion suppression mechanism sample was tested by Raman spectrometry to measure the residual stress between the MEMS S&A device and the energy-absorbing material. In summary, a reliable explosion suppression mechanism capability of the MEMS S&A device was achieved. The appearance of the MEMS S&A after the denotation wave output (Figure 15) shows that the MEMS S&A device integrated four types of energy-absorbing materials with a reliable explosion suppression mechanism capability.

Raman microscopy measurements of the residual stress on the MEMS S&A device surface are shown in Figure 15 from the Raman spectral peak shifts and the residual stress calculation formula:

(4) $$\sigma \;=-\;435\left(x_{test}-\;520\right)$$

where σ is the residual stress and x*_test_* is the relative strength of the surface of the MEMS S&A device. For systems under equivalent pressure, the optimum residual stress of the anodic bonding of different bonding voltages can be obtained. The residual stress were calculated for our energy-absorbing materials, and are given in Table 7.

The residual stress on the surface of the MEMS S&A device after the explosion suppression mechanism wave event for the various energy-absorbing materials is arranged in descending order as silver > nickel > copper > PI film. 

The Young's modulus of the silicon-based S&A device is close to the Young's modulus of the PI film. Therefore, in the same detonation field, the relative deformations of the two materials are similar. After the detonation wave is transmitted, the residual stress between the two materials is minimal, which reduces the secondary energy accumulation on the surface of the silicon-based S&A device, thus improving the silicon-based S&A device explosion suppression mechanism performance.

These results indicate that the PI film can achieve a reliable explosion suppression mechanism capability for a MEMS S&A device.

## 6. Conclusions

The traditional silicon-based MEMS S&A device fuze cannot isolate abnormal output in an explosion suppression mechanism environment. Based on this MEMS S&A device fuze, we designed a MEMS S&A device that integrates a silver, copper, nickel or PI film as an energy-absorbing material. First, we optimized the MEMS S&A device using theoretical calculations of the explosion suppression mechanism performance in the explosion suppression mechanism field, where the model was verified by dynamic simulations (LS-Dyna). Second, we used the silicon-based MEMS processing technology to integrate the MEMS S&A device with the energy-absorbing materials. Using the explosion suppression mechanism test, we compared the results from silicon-based MEMS S&A devices integrated with silver, copper, nickel and PI (100-μm-thick), which achieved a reliable explosion suppression mechanism capability under a detonation wave. The residual stress was measured by using Raman microscopy, and the results showed that the PI film exhibited the best explosion suppression mechanism performance of the four materials. A reliability test to determine the maximum explosion suppression mechanism dose for a MEMS S&A device attached to a PI film (100-μm-thick) was studied, where the maximum amount of primer needed for an effective explosion suppression mechanism capability on the MEMS S&A device was 0.45 mg.

## Figures and Tables

**Table 1:** Material parameters of glass, silicon wafer and explosion suppression mechanism energy-absorbing layer materials of Ag, Cu, Ni and polyimide (PI).

Material	ρ/(kg/m^3^)	Modulus of Elasticity E/GPa	Poisson’s Ratio ν	Breaking Strength/MPa
Si	2330	182	0.27	726
Glass	2550	88	0.16	480
Ag	10,500	732	0.2	605
PI	1420	200	0.37	1.1
Cu	8960	1230	0.34	315
Ni	8900	2010	0.31	515

**Table 2:** Values for the material parameters of the copper azide primer and the material pressure state equation constants in the explosion suppression mechanism field.

Parameter	Value	Parameter	Value
Density *ρ*/(g/cm^3^)	2.29	*A*/100 Gpa	4.1
Explosion Suppression Mechanism speed *D*/(cm/μs)	0.47	*B*/100 Gpa	0.045
Explosive pressure *P_CJ_*/100 GPa	0.126	*R*_1_	4.9
Initial relative volume *V*_0_	1.0	ω*	0.3
Initial ratio *E*_0_/100 GPa	0.085	*R*_2_	1.3

**Table 3:** Dimensions and corresponding quality parameter of the parameterized copper azide primer.

Parameter	Value				
Size (mm)	Φ2.5 × 1.47	Φ2.5 × 1.17	Φ2.5 × 0.87	Φ2.5 × 0.75	Φ2.5 × 0.6
Quality (mg)	10	7.989	5.94	5.121	4.097
Size (mm)	Φ2.5 × 0.375	Φ2.5 × 0.1875	Φ2.5 × 0.09375	Φ2.5 × 0.07032	Φ2.5 × 0.04688
Quality (mg)	2.56	1.28	0.64	0.4802	0.3201

**Table 4:** Parameters of the electroplated Cu metal.

Electroplated Solution	CuSO_4_	Coating Thickness	100 μm
Electroplated solution concentration	190 g/L	Stirring frequency	40 times/min
Current density	2.9 A/dm^2^	Electroplated cycle rate	8 L/min
Voltage	2.8 V	Time	20 min

**Table 5:** Patterned process of metal (copper).

Corrosion	Proportion Ratio (Volume)	Corrosion Rate	Horizontal Drill Rate
CH_3_COOH:H_2_O_2_:H_2_O	1:1:20	35 nm/min	19.8 nm/min

**Table 6:** Parameters used for the polyimide film spin coating process.

	Rotating Speed (r/s)	Accelerometer (r/s^2^)	Time (s)
Process1	500	500	10
process2	1000	500	10
process3	1500	500	10
process4	2000	500	10
process5	2500	500	10
process6	3000	500	30

**Table 7:** Raman spectral peak results and the surface residual stress of the MEMS S&A device for Ni, Cu and Ag metal films and polyimide (PI) film.

Adhesive Material	Frequency Shift Peak (cm^−1^)	Peak Width (cm^−1^)	Residual Stress (MPa)
Ni	519.547	4.79463	197.055
PI	520.073	5.36339	31.755
Cu	519.598	4.94299	174.87
Ag	519.511	4.79051	212.715
